# Statolith Morphometrics Can Discriminate among Taxa of Cubozoan Jellyfishes

**DOI:** 10.1371/journal.pone.0155719

**Published:** 2016-05-18

**Authors:** Christopher J. Mooney, Michael J. Kingsford

**Affiliations:** College of Marine and Environmental Sciences and ARC Centre of Excellence in Coral Reef Studies, James Cook University, Townsville, QLD, Australia; University of Connecticut, UNITED STATES

## Abstract

Identification of potentially harmful cubomedusae is difficult due to their gelatinous nature. The only hard structure of medusae, the statolith, has the potential to provide robust measurements for morphometric analysis. Traditional morphometric length to width ratios (L: W) and modern morphometric Elliptical Fourier Analysis (EFA) were applied to proximal, oral and lateral statolith faces of 12 cubozoan species. EFA outperformed L: W as L: W did not account for the curvature of the statolith. Best discrimination was achieved with Canonical Discriminant Analysis (CDA) when analysing proximal + oral + lateral statolith faces in combination. Normalised Elliptical Fourier (NEF) coefficients classified 98% of samples to their correct species and 94% to family group. Statolith shape agreed with currently accepted cubozoan taxonomy. This has potential to assist in identifying levels of risk and stock structure of populations in areas where box jellyfish envenomations are a concern as the severity of envenomation is family dependent. We have only studied 12 (27%) of the 45 currently accepted cubomedusae, but analyses demonstrated that statolith shape is an effective taxonomic discriminator within the Class.

## Introduction

Cubozoans are a class within the phylum Cnidaria. Collectively known as the box jellyfishes, there are currently 45 accepted species of cubomedusae worldwide split into the two orders of Carybdeida (32 species) and Chirodropida (13 species; [1]). Many more species remain undescribed and this total is expected to rise [2]. The taxonomy of this class is largely based on the morphology of soft parts: carybdeids have a single tentacle per pedalium with most having nematocysts present on both tentacle and bell; chirodropids generally have multiple tentacles per pedalium with nematocysts usually found only on tentacles. Due to their gelatinous nature and rarity of many species identification can be difficult and taxonomy of this class is regularly updated [3,4,5,6,7,8,9].

Cubomedusae are perhaps best known for their harmful and potentially fatal stings to humans. Well known in Australia, envenomations from cubozoans have received more global interest in recent years (e.g. [10,11,12,13,14]). Within Australia, box jellyfish envenomations of concern are due to two main syndromes: the immediately painful and potentially fatal envenomation from *Chironex fleckeri* (responsible for approximately 70 fatalities in Australia [15,16]) or the delayed onset of debilitating symptoms associated with Irukandji syndrome (attributed to two deaths in Australia [17], at least one of which was confirmed as *Carukia barnesi* [18]). On the Great Barrier Reef cubozoans are considered one of the greatest threats to tourism [2] with Irukandji syndrome attributed to over $65 million loss to the industry due to negative publicity in 2002 [19]. Stings from Irukandji jellyfishes have been named as the number one occupational health and safety issue for Australia’s pearling industry, and beche de mer and tropical lobster fisheries [10]. As not all cubozoans are equally venomous to humans, reliable identification of box jellyfishes is a critical issue for risk management [6]. Sting-related concerns are not unique to Australia, cubozoans are found throughout the tropical world and affect people’s lives at multiple latitudes and longitudes.

Identification of cubomedusae has historically been achieved using distinctive structural characters. For Carybdeida, phacellae arrangements, rhopalial niche, or tentacle form have been listed as the most useful characters for distinguishing species [3]. For Chirodropida, the form of gastric saccules [20], pedalial canal and branching, lateral gonads, and body and tentacle size and shape have been the most useful for taxa discrimination [4]. However, all of these structures are soft, pliable, and fragile gelatinous construct of the medusa form. Identification using soft tissue characters can be problematic as bell and tentacle tissue can easily be damaged depending on method of collection; specimens found washed up on a beach may be partially decomposed or even fragmentary following a predation event; and they can be difficult to recognise if animals are preserved in ethanol for genetic assays. Cubomedusae do however have a hard structure: the statolith.

**Fig 1:**
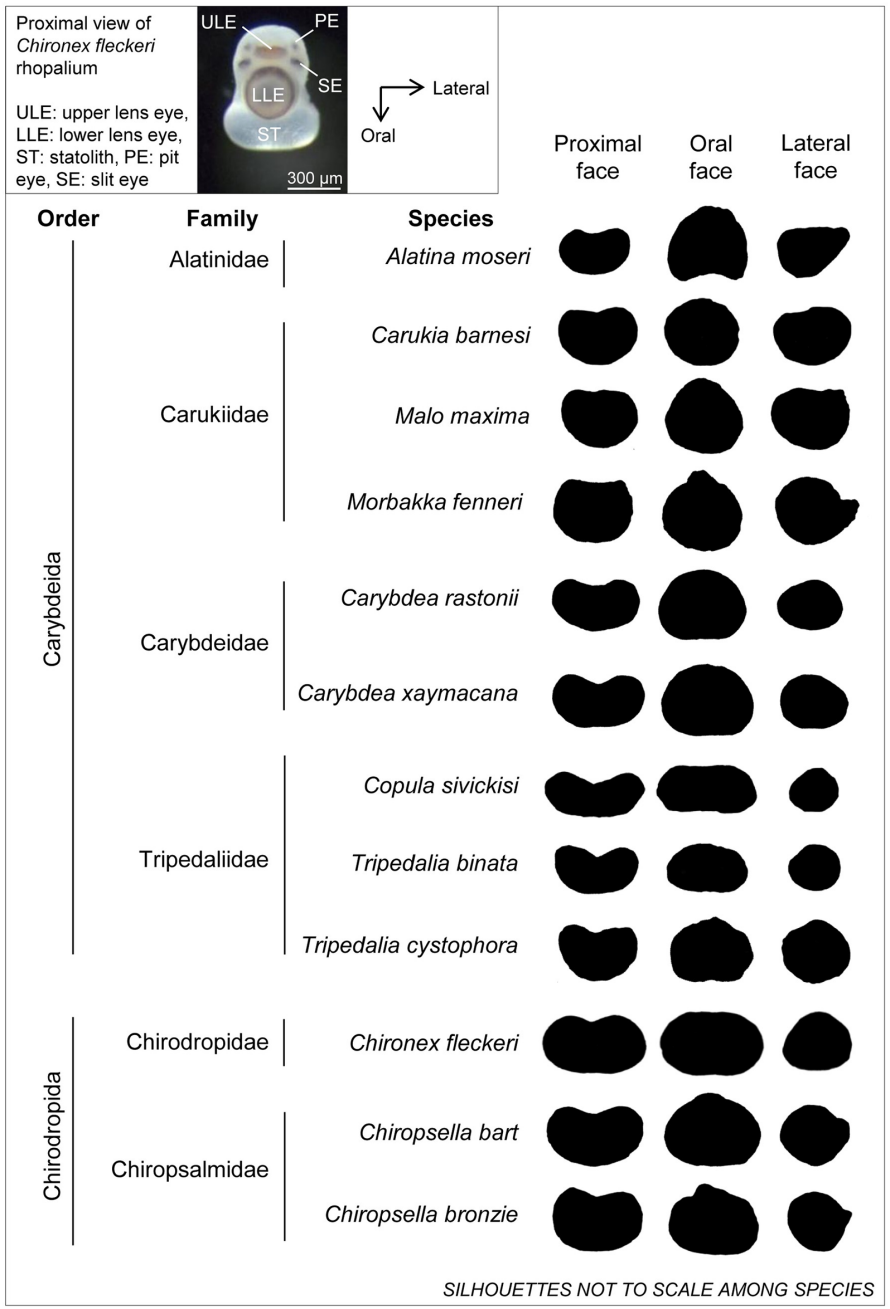
Proximal view of *Chironex fleckeri* rhopalium showing location and orientation of statolith (inset) and proximal, oral and lateral face silhouettes of a single random example statolith for 12 cubozoan species.

Cubomedusae possess statoliths to orientate their sensory organs which are used to orientate the entire medusae. The statolith, a hard, crystal-like structure, resilient to easy deformation, forms within the statocyst membrane in the distal part of the rhopalium which holds the eyes (inset Fig 1). As the only hard structure of cubomedusae the statolith provides an opportunity for robust morphometric analysis. Otolith and statolith shapes have been found to be distinctly species specific in fishes (e.g. [21,22]) and cephalopods (e.g. [23,24]); accordingly, they have proved valuable tools for taxonomy and ecology in these taxa. Ontogenetic changes in the shape of fish otoliths and cephalopod statoliths can confound among species comparisons as structures can be of similar shape during developmental stages and differentiate more in shape with age [25,21]. Statoliths of the closely related scyphomedusae, who possess sacks of numerous small statoliths, have also been found to have differences in shape among some species [26]. Similar to scyphozoans, newly metamorphosed cubomedusae possess groups of many small statolith crystals within statocyst membranes [27,28]. These combine and form discrete, large single statoliths as the medusa grows and adult cubozoan statoliths may provide a robust taxonomic tool; qualitative assessments have suggested that cubozoan statolith shape may be genus specific [14]. However, qualitative descriptions are imprecise, and can have limited powers of discrimination given differences in shape among taxa can be complex and subtle without detailed measurements [29].

Analyses of shape have been used widely to describe phenotypic variation among and within taxa. Shape can be defined as “the total of all information invariant under translations, rotations and isotropic rescaling” [30,31]. For morphometric analyses there is a requirement that variation in shape be represented in describable and repeatable terms [32]. A quantitative framework, providing a rigorous method of describing shape, was introduced by traditional morphometrics [29]. Traditional morphometrics mostly relies on the collection of raw linear measurements and typically applies statistical methods to distances and distance ratios, areas, volumes and angles [33,34]. Geometric morphometrics advanced on this and is the analysis of Cartesian geometric coordinates of morphological structures rather than linear, areal or volumetric variables [35]. Several techniques exist in modern geometric morphometrics including truss networks, superimpositions, thin plate splines and analyses of outlines (see [36]). Elliptical Fourier Analysis (EFA) of outlines has proved to be a reliable tool to analyse and compare subtle variations in shape for a wide range of applications, including terrestrial (e.g. pollen grains [37] and weed and crop plant species [38]) and marine taxa (e.g. fish otoliths [39], octocoral sclerites [40] and shells [41]).

The objective of this study was to use statolith shape to discriminate among adults from taxa of the class Cubozoa. Our approach was to use the proximal, oral and lateral faces of statoliths to test for differences among taxa. The specific aims were to (1) discriminate among taxa using traditional morphometrics of length: width ratios; (2) discriminate among taxa using the modern approach of EFA; and (3) determine if statolith shape is dependent on statolith size.

## Materials and Methods

### Samples

A combination of archived, wild caught and laboratory cultured specimens of 12 separate species were used in this study, ~ 30% of known cubozoan diversity; ten carybdeids and three chirodropids (see Table A in S1 File). Carybdeids included: *Alatina moseri* Mayer 1906 (Alatinidae), *Carukia barnesi* Southcott 1967, *Malo maxima* Gershwin 2005, and *Morbakka fenneri* Gershwin 2008 (Carukiidae), *Carybdea rastonii* Haacke 1886 and *Carybdea xaymacana* Conant 1897 (Carybdeidae), *Copula sivickisi* Stiasny 1926, *Tripedalia binata* Moore 1988 and *Tripedalia cystophora* Conant 1897 (Tripedaliidae). Chirodropids included: *Chironex fleckeri* Southcott 1956 (Chirodropidae) and *Chiropsella bart* Gershwin and Alderslade 2007 and *Chiropsella bronzie* Gershwin 2006 (Chiropsalmidae).

All medusae were collected from Australian waters with the exception of *Tripedalia cystophora* which were raised from a cultured polyp stock in laboratory conditions (provided by Anders Garm, University of Copenhagen, Denmark; originally sourced from Puerto Rico). Specimens collected within the Great Barrier Reef Marine Park were collected under the Great Barrier Reef Marine Park Authority (Marine Parks Permits G08/27913.1 and G11/34552.1). No specific permissions were required for any other collection locations/ activities as the species involved are not endangered or protected and other collection sites did not require permits.

Wild medusae were attracted to night-lights and collected with scoop nets, some were collected with beach seine nets, by hand or by routine drag netting of patrolled beaches by Surf Life Saving Queensland. Medusae were identified from descriptions of adult medusa using bell/ tentacle morphology and preserved in 100% ethanol. Ethical approval was not required for research on medusae. Following extraction from the rhopalial niche, statoliths were separated from rhopalia using needles under a dissecting microscope and stored in 100% ethanol. It should be noted that statoliths will deteriorate if samples are preserved in formaldehyde.

### Obtaining statolith images

One statolith per medusa was used for analyses as it was determined one statolith is representative of the other three in a medusa [42]. A section of black electrical tape on a glass slide had a minute amount of glue smeared onto it which allowed for temporary adhesion. The statolith was placed in this temporary adhesion orientated proximal face up (cleavage vertical; see Fig 1). The statolith was illuminated using a cold lamp source then photographed and measured using Leica IM50 software (Version 4.0 Release 132, Leica Microsystems Imaging Solutions Ltd. 2004) coupled with a Leica DC300 camera fitted to a Leica DMLB microscope. The same procedure was then followed taking images of the statolith oral (cleavage down) and lateral (cleavage horizontal) faces (Fig 1). Greyscale statolith images including a 100 μm scale bar were then inverted and some minor editing done (removal of statocyst membrane fragments that were not sulphate material, or local blurring, after consulting original image) using Adobe Photoshop CS5.1 leaving a black statolith silhouette on white background in Tagged Image File Format (TIFF).

### Length: Width ratios

Statolith silhouette images were imported into R 3.0.2 [43] and calibrated to scale using the locator function of the graphics package (see Appendix A in S1 File for details and R script). A comparison of Length: Width ratios (L: W) measured using R to values measured using Leica IM50 software found a mean error of -1.29% (*n* = 117 images) which suggested that using the locator and ild [36] functions in R resulted in acceptable L: W values.

### Elliptical Fourier Analysis

Silhouette images were converted to.jpg files so that they could be used in the Momocs [44] package in R. Silhouettes were then imported into R and statolith outlines were extracted into a ‘Coo’ class object as a list of x; y pixel coordinates from the black silhouette on white background by Momocs using an algorithm implemented in R by Claude ([36]; see Appendix B in S1 File for script). Elliptical Fourier Analysis (EFA) was then performed on statolith outlines. Detailed descriptions of this method are well documented elsewhere (see [36,29]). The basis of EFA is to separate the x and y coordinates of the outline and calculate discrete Fourier series on these two periodic functions using a number *n* of harmonics high enough to capture a satisfactory amount of the geometry of the shape described [37]. Multivariate analysis can then be performed on the 2*n* harmonic coefficients obtained for each shape. Although some species required on average < 5 harmonics to describe > 99% of statolith proximal face shape (e.g. *Carybdea rastonii*, *Copula sivickisi*), 18 harmonics was the maximum *n* required of any species or statolith face analysed here to describe > 99% of its shape (see Table B in S1 File). As such, to encompass as much variation in shape as possible, EFAs on all silhouettes were performed on 20 harmonics with 300 outline smoothing iterations.

Parameters of the first harmonic were used to normalise coefficients so that they were invariant to size, rotation and starting point of the outline trace (default for Momocs EFA). This resulted in the Normalised Elliptic Fourier (NEF) coefficients A_*n*_, B_*n*_, C_*n*_ and D_*n*_. When normalising to the first harmonic A_1_, B_1_, and C_1_ become constant and D_1_ is concerned with the relative dimensions of the first ellipse and is often subject to error measurement dependent on orientation [36]. NEF harmonic 1 was thus omitted and further analyses were conducted on NEF coefficients for harmonics 2–20 (76 variables).

### Statistical analyses

Statistical analyses were conducted in SYSTAT 11 (SYSTAT Software, Inc. 2004). The model that statolith L: W ratios would be consistent among species was tested separately for proximal, oral and lateral faces with one-way Analysis of Variance (ANOVA). Tukey’s HSD pairwise means comparison post hoc test was used to compare differences among species. Canonical Discriminant Analysis (CDA) with the addition of a ‘leave one out’ jackknife classification cross validation was then performed on L: W of all statolith faces (proximal + oral + lateral) to test for better discrimination amongst taxa using a three-dimensional approach to statolith shape.

In the ‘leave one out’ jackknife procedure with a subset of *n* observations (x_1_, x_2_, …..x_*n*_), *n* subsets are chosen by omitting each observation in turn and then repeating the CDA on each subset. Correct classification post jackknifing is then computed from the results of these *n* analyses (SYSTAT Software Inc., 2004).

Principal Component Analysis (PCA) was used to determine which NEF coefficients explained most variation in statolith shape. PCAs were performed separately on proximal, oral and lateral statolith face NEF coefficients to produce a reduced data set with best discriminatory power. Coefficients with eigenvalues > 1 were maintained and those with eigenvalues < 1 were removed from further analyses as they were thought to add little to the explanation of variation. CDAs with a ‘leave one out’ jackknife classification cross validation were then performed on the reduced data set separately for proximal, oral and lateral faces and then on possible combinations of statolith faces: proximal + oral, proximal + lateral, oral + lateral, and proximal + oral + lateral to determine which was best for species discrimination. CDA on proximal + oral + lateral faces was then repeated with samples grouped by family not species.

Least squares regression was applied to test the dependence of statolith shape (average of all NEF coefficients) on statolith size (statolith length). Regressions were performed separately for each species.

## Results

### Length: Width ratios

**Fig 2:**
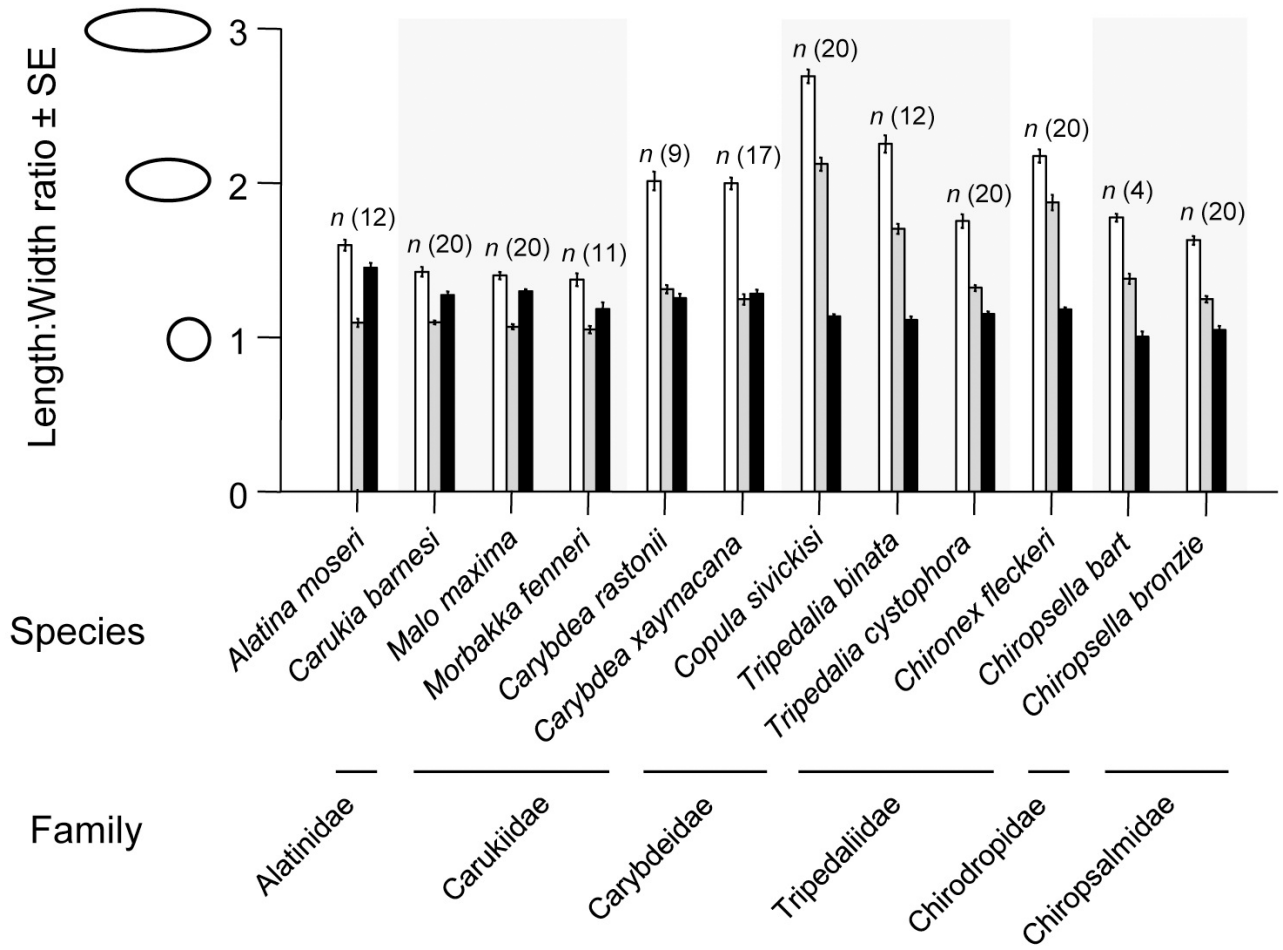
Mean Length: Width ratios for statolith proximal face (white), oral face (grey) and lateral face (black) for 12 cubozoan species. ANOVA—Proximal face: *F*_(11, 173)_ = 109.832, *p* < 0.001; Oral face: *F*_(11, 173)_ = 133.929, *p* < 0.001; Lateral face: *F*_(11, 173)_ = 21.428, *p* < 0.001. *(See* Table C in S1 File *for post-hoc results)*.

There were clear qualitative differences in shape among taxa (Fig 1). Furthermore, statolith L: W ratios varied among species and this was significant for all statolith faces (Fig 2). Higher L: W resembled longer, thinner profiles (characteristic of samples from Carybdeidae, Tripedaliidae and Chirodropidae) and lower L: W resembled more spherical shapes (characteristic of samples from Carukiidae; Fig 1). Among statolith faces L: W ratios of proximal faces were greatest for all species, yet ranking between oral and lateral face ratios were family dependent. Some families showed greater lateral L: W than oral L: W (Alatinidae and Carukiidae); some showed greater oral than lateral L: W ratios (Tripedaliidae, Chirodropidae and Chiropsalmidae); and Carybdeidae showed very similar oral and lateral L: W ratios (Fig 2).

*Copula sivickisi* had one of the most distinguishable statolith shapes (Fig 1) and showed the highest L: W for proximal and oral faces, significantly differing from all other species, yet its lateral face L: W was similar to seven other species (Fig 2, Table C in S1 File). The lateral L: W of *Alatina moseri* was significantly distinct among the species, yet it shared similar proximal and oral L: W with four other species (Figs 1 and 2, Table C in S1 File). Some statolith faces showed similar L: W ratios between some species (e.g. *Chironex fleckeri* proximal L: W was similar to *Copula sivickisi* oral L: W; and *Alatina moseri* proximal L: W was similar to *Tripedalia binata* oral L: W; Figs 1 and 2).

It was clear that statolith L: W ratios were similar among species within a family (Fig 2) with non-significant differences found among species within Carukiidae, Carybdeidae and Chiropsalmidae. This was found for proximal and oral faces and also included Tripedaliidae for lateral face L: W (see Table C in S1 File).

**Fig 3:**
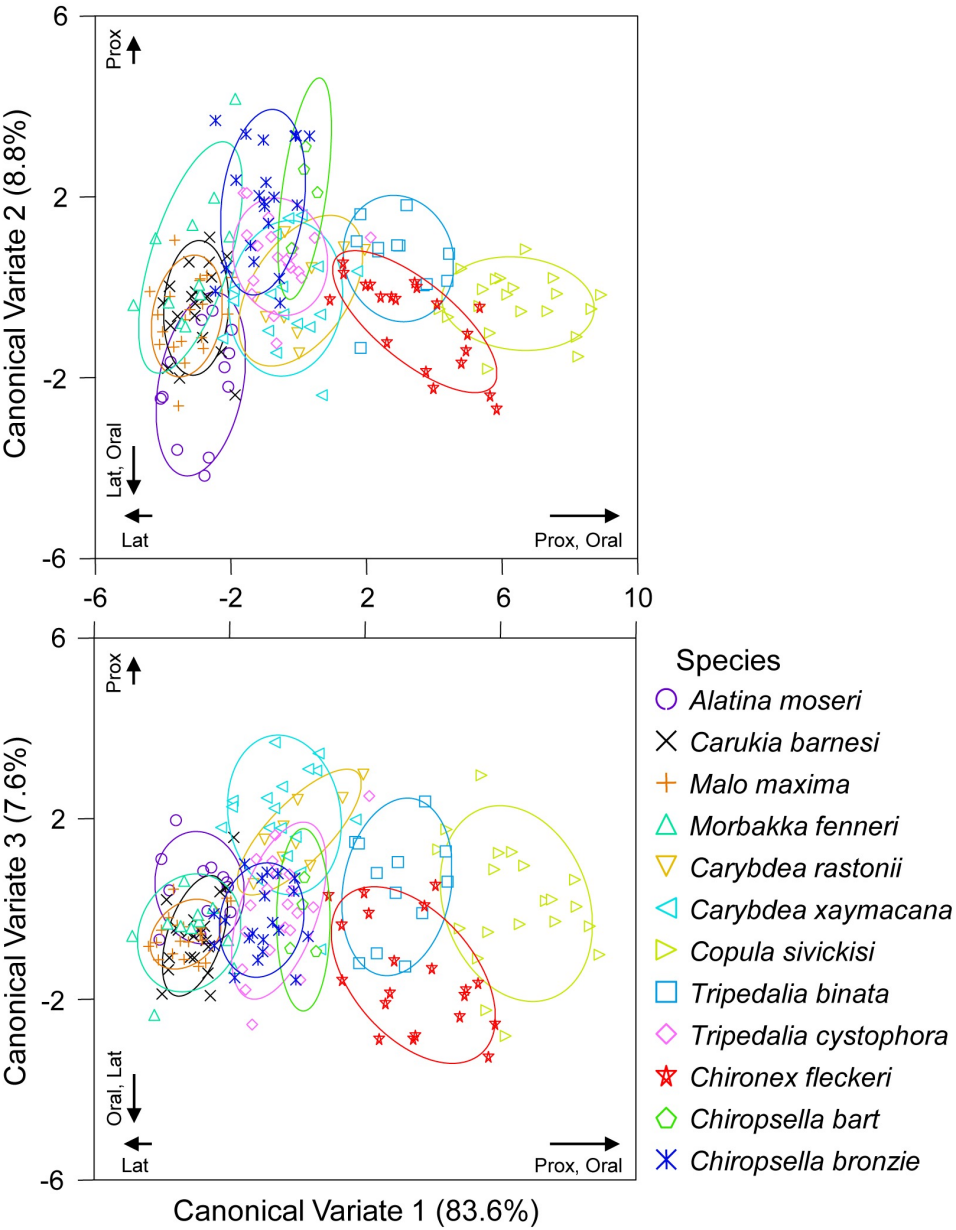
Canonical Discriminant Analysis of statolith Length: Width ratios for proximal + oral + lateral faces among species. Ellipses = confidence around group data. Individual statolith face loadings [Proximal (Prox), Oral, Lateral (Lat)] are indicated for each CV.

A multivariate comparison of statolith proximal + oral + lateral L: W found L: W could be used to successfully distinguish among some species (CDA; Wilk’s lambda = 0.021, *F*_(33, 504)_ = 41.404, *p* < 0.001). There was however, considerable overlap of other species (Fig 3). A high percentage of correctly classified statoliths were found for *Tripedalia binata* (92%), *Copula sivickisi* (85%), *Alatina moseri* and *Chironex fleckeri* (75%), *Carybdea xaymacana* (71%) and *Chiropsella bronzie* (70%). *Tripedalia binata* (83%), *Copula sivickisi* (80%), *Carybdea xaymacana* (71%) and *Chironex fleckeri* (70%) maintained high classification success following jackknifed classification (Fig 3, Table D in S1 File). A high proportion of misclassification of samples occurred within families (e.g. Carukiidae; Table D in S1 File).

### Elliptical Fourier Analysis

Of 76 possible NEF coefficients 28 had eigenvalues > 1 for proximal face, 29 for oral face and 26 for lateral face, these described most of the variance in shape.

**Fig 4:**
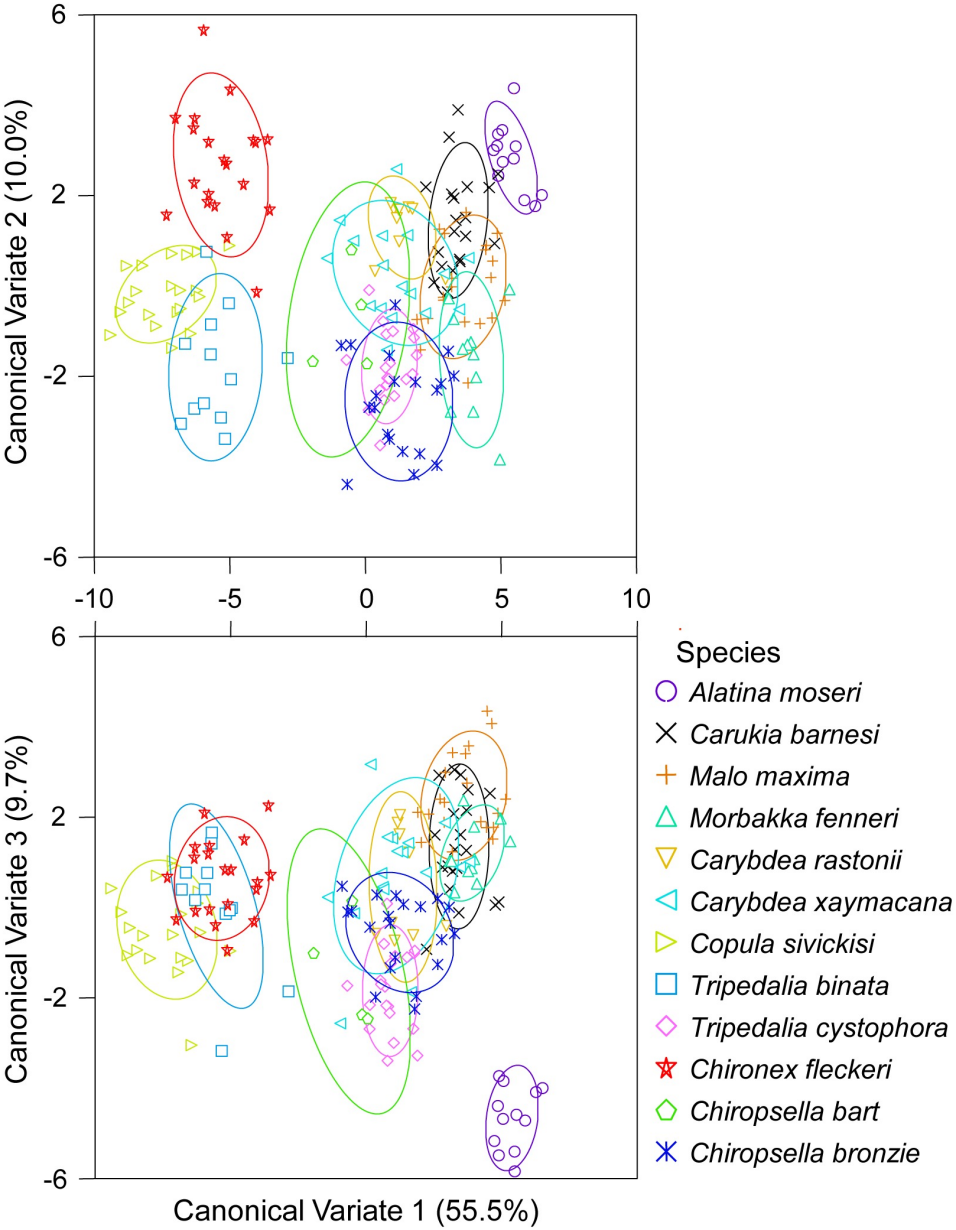
Canonical Discriminant Analysis of Normalised Elliptical Fourier coefficients for proximal + oral + lateral statolith faces among species. Ellipses = confidence around group data.

A CDA on proximal + oral + lateral faces returned the highest correct classifications, successfully distinguishing among species (Wilk’s lambda = 0.000, *F*_(913, 1033)_ = 1.996, *p* < 0.001), CDAs of separate faces or other face combinations (i.e. proximal + oral, proximal + lateral, or oral + lateral faces) resulted in lower resolution. From the three face comparison 10 of the 12 species studied had 100% of samples correctly classified to them and the remaining two species, *Chiropsella bronzie* and *Carybdea xaymacana*, had 95% and 82% of samples correctly classified respectively (Fig 4, Table 1). Following jackknifed classification three species had ≥ 65% of samples correctly classified to them: *Chironex fleckeri* (90%), *Copula sivickisi* (85%) and *Carukia barnesi* (65%; Table 1). Again a large proportion of misclassifications following leave one out cross validation could be seen within families (e.g. Carukiidae and Carybdeidae; Fig 4, Table 1).

**Table 1:** Canonical Discriminant Analysis classifications for Normalised Elliptical Fourier coefficients for statolith proximal + oral + lateral faces among species (bold = percent of samples correctly classified). Percentages (%) are rounded to whole numbers.

				Species classified to following Jackknifed classification											
				(% of samples)											
Family	Species	*n*	% correct classification	*Alatina moseri*	*Carukia barnesi*	*Malo maxima*	*Morbakka fenneri*	*Carybdea rastonii*	*Carybdea xaymacana*	*Copula sivickisi*	*Tripedalia binata*	*Tripedalia cystophora*	*Chironex fleckeri*	*Chiropsella bart*	*Chiropsella bronzie*
Alatinidae	*Alatina moseri*	12	**100**		33	8	17	8	8					17	8
Carukiidae	*Carukia barnesi*	20	**100**		**65**	10	5	5	5			5			5
	*Malo maxima*	20	**100**	5	35	**15**	10	5	15			5		5	5
	*Morbakka fenneri*	11	**100**			27	**18**	18						18	18
Carybdeidae	*Carybdea rastonii*	9	**100**		22		11	**11**	44			11			
	*Carybdea xaymacana*	17	**82**		18	12		24	**12**			12	6		18
Tripedaliidae	*Copula sivickisi*	20	**100**							**85**	10		5		
	*Tripedalia binata*	12	**100**							8	**50**	8	33		
	*Tripedalia cystophora*	20	**100**	10		5	5	10	15		5	**20**			30
Chirodropidae	*Chironex fleckeri*	20	**100**							5	5		**90**		
Chiropsalmidae	*Chiropsella bart*	4	**100**		25				25			25			25
	*Chiropsella bronzie*	20	**95**				10	5	15		5	10			**55**

**Fig 5:**
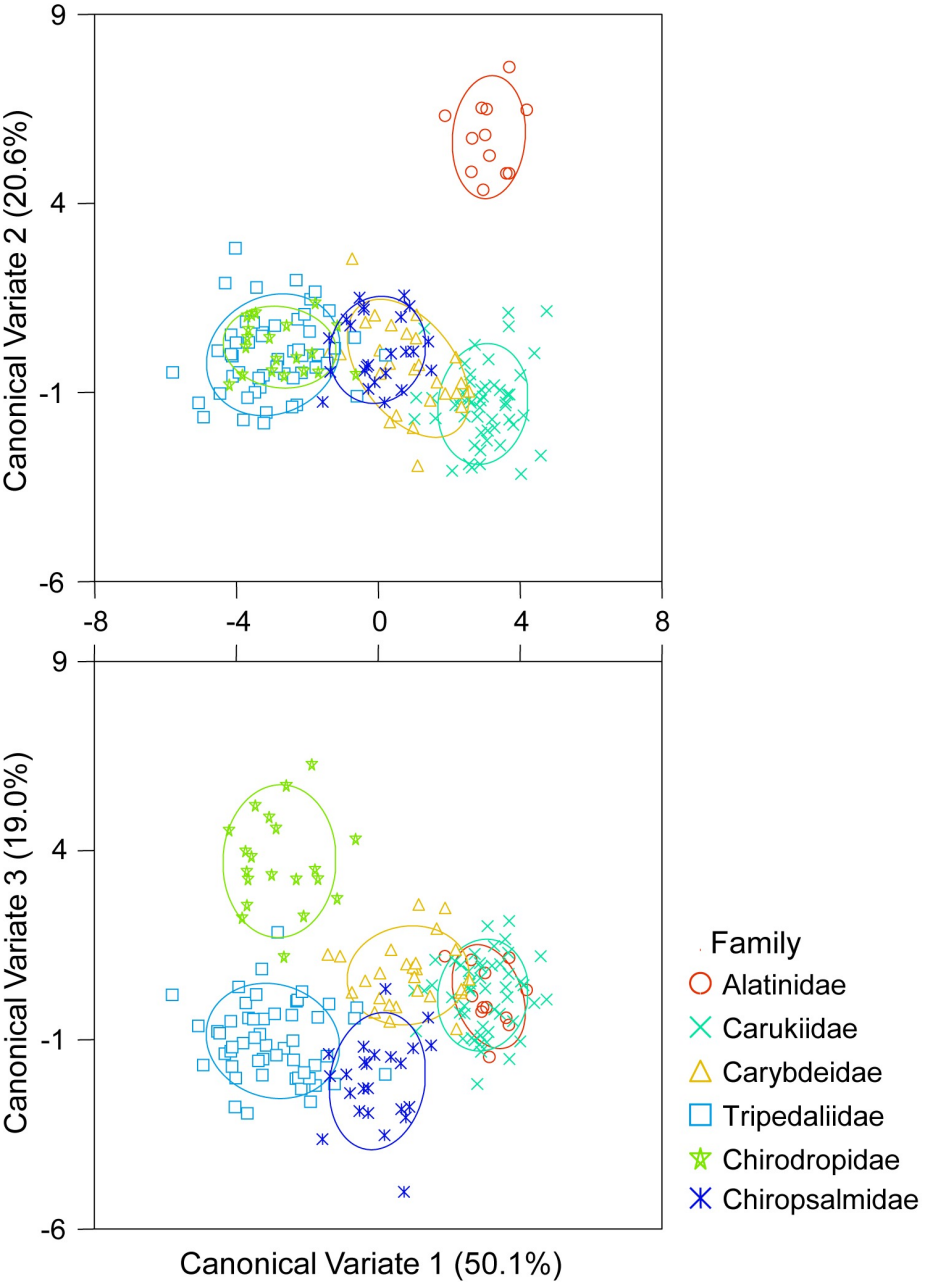
Canonical Discriminant Analysis of Normalised Elliptical Fourier coefficients for proximal + oral + lateral statolith faces among families. Ellipses = confidence around group data.

When grouped by family instead of species, CDA of proximal + oral + lateral faces successfully discriminated among families (Wilk’s lambda = 0.004, *F*_(415, 489)_ = 2.477, *p* < 0.001, Fig 5). 100% of samples were correctly classified to Alatinidae, with four families (Tripedaliidae, Chiropsalmidae, Chirodropidae and Carukiidae) showing > 90% correct classification, with Carybdeidae showing 81% correct classification (Table 2). Following jackknifing, two families maintained correct classification ≥ 50% [Chirodropidae (85%) and Carukiidae (53%), Table 2].

**Table 2:** Canonical Discriminant Analysis classifications for Normalised Elliptical Fourier coefficients for statolith proximal + oral + lateral faces among families (bold = percent of samples correctly classified). Percentages (%) are rounded to whole numbers.

			Family classified to following Jackknifed classification					
			(% of samples)					
Family	*n*	% correct classification	Alatinidae	Carukiidae	Carybdeidae	Tripedaliidae	Chirodropidae	Chiropsalmidae
Alatinidae	12	**100**	**8**	33	17		8	33
Carukiidae	51	**94**	10	**53**	25	2		10
Carybdeidae	26	**81**		42	**23**	15		19
Tripedaliidae	52	**98**	6	6	12	**48**	13	15
Chirodropidae	20	**95**			5	10	**85**	
Chiropsalmidae	24	**96**	4	13	25	17		**42**

Regression analysis demonstrated that statolith shape was not dependent on statolith size for most species (11 out of 12 studied; Table E in S1 File). *Malo maxima* were found to show significant dependence of statolith shape on size (Table E in S1 File). However, the ordination plot of CDA on proximal + oral + lateral faces showed that this had little influence on the assignment of samples to species groups (Fig 4).

## Discussion

We have clearly demonstrated, at least for the species studied here, that adult statolith shape is a robust and effective tool for discriminating families of cubozoans and in many cases individual species. This shows great potential for the entire class. Qualitatively this appeared likely (Fig 1), quantitatively L: W could distinguish among families and some species. Further, the modern method of EFA was most effective in distinguishing among all species studied. Ratioing of measurements removed the effect of variation in size and had the advantage of simple computation and being easily interpreted in geometric terms of shape variation [36]. Although it removes the size parameter, using ratios can have the disadvantage of increasing correlations between variables because data becomes dependent after being standardised [36]. L: W was limited as the most basic of shape estimates; it did provide information on long and thin versus short and fat yet it gave no account of the curvature of the statolith. EFA clearly outperformed L: W in correctly classifying samples to their species group, and successful classification increased with the number of statolith faces included in analyses, with best results seen when combining proximal + oral + lateral faces. Williams *et al*. [45] also recently compared L: W and EFA for discriminating among seed shapes of wheat. They too found that EFA was better for shape description than L: W direct measurements, stating that a seed’s crease depth variation was not described using only direct measurements of major axes of seed dimensions.

It is critical to define an object’s orientation when comparing shape. When viewing a cubozoan statolith from an undefined angle it could be easy to see similarities in shape between species. When looking at the defined proximal, oral and lateral faces separation among species becomes easier. It is important to assess all three faces as separation may not be capable if only looking at one or two. For example, no significant difference was found between *Alatina moseri* and *Carukia barnesi* statoliths for proximal and oral faces using L: W, yet lateral face could significantly distinguish the two (Table C in S1 File). The importance of orientation has also been highlighted elsewhere. Neto *et al*. [38] noted two angles were needed to describe a leaf plane in three-dimensional (3D) space and Williams *et al*. [45] similarly noted that best description of seed shape came from 3D assessments using two images in different orientations (horizontal and vertical projections); it was concluded that differences in shape could go undetected if only using one two-dimensional image.

The use of proximal + oral + lateral statolith faces resulted in an average of 98% correct classification of samples to species groups overall. However, this dropped to an average of 42% following the leave-one-out jackknifed cross validation. A common problem with many morphologic studies using multivariate statistics is potentially inadequate sample sizes [46]. False conclusions regarding differences among groups could result from small sample sizes failing to capture the covariance and morphological variation adequately [47]. Most cubomedusae are rare, and collection of large sample sizes even more so. Species which maintained high correct classification following leave-one-out jackknifed cross validation all had a sample size = 20 (e.g. *Chironex fleckeri*; 90%, *Copula sivickisi*; 85%, and *Carukia barnesi*; 65%). Those species with low sample sizes (*n*) typically had low correct jackknifed classification (e.g. *Chiropsella bart* (*n* = 4; 0%), *Alatina moseri* (*n* = 12; 0%), and *Carybdea rastonii* (*n* = 9; 11%)). Despite low sample sizes for many species, CDA correctly classified 100% of samples to 10 out of the 12 species studied (Table 1). Clearly adult statolith shape is of high utility; many taxa are very rare (e.g. [48]) but for the samples studied here the probability of accurate identification to family, and in some cases species was very high.

High levels of misclassification of samples were seen among species within families. This was apparent in both L: W and EFA. Qualitatively, similarities among species within families could easily be seen (Fig 1). Further, quantitatively even L: W of all three faces easily depicted similarities in statolith shape within families (Fig 2). Although the L: W approach was not as successful as EFA at differentiating species groups, this simple approach could be applied to quickly assign a statolith to it’s likely family. Statolith shape similarities among species within families, and differences among families, was also easily visualised from sample groupings in the CDA of proximal + oral + lateral NEF coefficients (Figs 4 and 5).

Differences in statolith shape supported the currently accepted taxonomy of Cubozoa [1]. *Alatina moseri* (Alatinidae) was distinct, being well separated from other families. All three species of Carukiidae were tightly grouped. *Carybdea rastonii* and *Carybdea xaymacana* showed considerable overlap with each other and Carybdeidae is a distinct group. Statolith shape showed that *Copula sivickisi* was well grouped within Tripedaliidae and supported the recent move of this species from the Carybdeidae to Tripedaliidae [5]. The grouping of statolith samples also easily distinguished members of the Chirodropida, clearly separating the Chirodropidae and Chiropsalmidae.

Statolith shape is clearly a useful tool for assisting in the identification of cubomedusae. As the only hard structure of box jellyfishes, this would likely have best utility in specimens damaged or fragmented during collection (if rhopalia are intact), or ethanol preserved specimens. This can be useful for risk management as the shape of the statolith can identify the relative danger of box jellyfish to humans as the severity of envenomation differs between families. Within the order Carybdeida, envenomation from species of Alatinidae and Carukiidae are known to produce Irukandji syndrome whereas envenomation from Carybdeidae is circumstantial and envenomation from Tripedaliidae does not produce Irukandji syndrome (see [5,14]). Within the order Chirodropida, envenomations from species of Chirodropidae are known to be fatal [2] whereas envenomation from Chiropsalmidae, while painful, are far less harmful. This will certainly assist in risk management in areas such as northern Queensland, Australia where morphologically very similar medusae are commonly found in the same location. For example, *Carukia barnesi* (typical Irukandji syndrome jellyfish linked to at least one fatality [18]) and *Carybdea xaymacana* (speculative as to whether or not responsible for severe Irukandji syndrome [14]), or *Chironex fleckeri* (potentially fatal, linked to almost 70 deaths in Australia [16]) and *Chiropsella bronzie* (non fatal [4]).

In addition to species specific differences, fish otolith shape can often vary geographically within a species [49]. EFA of otolith silhouettes has been found to be successful in finding regional and site specific shape differences in several fish species (e.g. [50,51,52,53,54]), proving an invaluable tool for stock management. As statolith shape has been proven to be robust for morphometric analyses here, the technique shows potential to elucidate much needed knowledge on cubomedusae population structure. However, it would likely be dependent on large sample sizes.

Allometric change can be an issue when using body structures for identification. Hecht and Appelbaum [21] found that the otolith shape of deep-sea eels was only robust for identification of adult specimens as early stages of otolith formation were indistinguishable. Changes in squid statolith shape were also noted as squid matured, and this was linked to changes in habitat ecology of pelagic versus near bottom [55]. Regression analysis found cubozoan statolith shape was not dependent on statolith size in all species studied except *Malo maxima* (Table E in S1 File). *Malo maxima* showed significant dependence of statolith shape on statolith size, perhaps suggesting that the specimens studied here covered a range of developmental stages, both immature and mature medusae for this species. However, variation in the size of statoliths did not affect classification of *Malo maxima* samples to their correct species (Fig 4, Table 1). Over at least the size range of medusae/ statoliths analysed (see Table A in S1 File), adult cubozoan statolith shape is a useful tool for identification regardless of size.

## Conclusions

Variation in adult cubomedusae statolith shapes studied here was family specific and in many cases species-specific. It was demonstrated that there was significant discrimination from both traditional L: W and modern EFA morphometric techniques among the studied taxa. EFA outperformed L: W in correct classification of statolith shape to species as L: W gave no account of curvature of the statolith. Best results were seen when statolith proximal, oral and lateral faces were analysed in combination, with CDA of NEF coefficients resulting in 98% correct classification overall of samples to species and 94% overall to family. Statolith shape of the species studied here agreed with currently accepted taxonomy and can be used to quickly assign adult specimens to at least family by basic L: W of statolith faces. This has great potential to assist in risk management and the determination of stock structure in areas where box jellyfish envenomations are a concern as the severity of envenomation is family dependent. We studied 12 of the 45 currently accepted cubomedusae; our comparison encompasses ~ 30% of taxa and demonstrates that statolith shape is a useful additional taxonomic tool for the Class. The statoliths of cubomedusae provide the only hard structure that can be used as a robust taxonomic tool especially where other diagnostic tools such as the soft bodied medusa or tentacles are damaged. Our findings demonstrate that variation in adult statolith shape among taxa is of high utility for studies on the taxonomy, phylogeny and ecology of cubozoans.

## Supporting Information

S1 File Supporting Info_Mooney & Kingsford, Cubozoan ID using statolith shape. Supporting Tables and R scripts for statolith morphometric analyses.
(DOCX)
