# Supplementary material for: Statolith Morphometrics Can Discriminate among Taxa of Cubozoan Jellyfishes
Source: PLoS One. 2016 May 18;11(5):e0155719. doi: 10.1371/journal.pone.0155719 (PMC4871450; doi:10.1371/journal.pone.0155719)
Supplement: S1 File — Supporting Tables and R scripts for statolith morphometric analyses. (DOCX) [file pone.0155719.s001.docx]

**Statolith morphometrics can discriminate among taxa of cubozoan jellyfishes**

**Supporting Information (S1)**

Christopher J Mooney, Michael J Kingsford

**S1 Table A.** Summary of the specimens used in the study. QLD; Queensland, SA; South Australia, WA; Western Australia, NT; Northern Territory, IPD; Interpedalial distance, Min.; Minimum, Max.; Maximum, ND; no data – archived statolith samples

|  |  |  |  |  | IPD (mm) | | | Statolith length (µm) | | |
| --- | --- | --- | --- | --- | --- | --- | --- | --- | --- | --- |
| Order | Family | Species | *n* | Collection site | Min. | Max. | Mean | Max. | Min. | Mean |
| Carybdeida | Alatinidae | *Alatina moseri* | 12 | Port Douglas, QLD, Australia | ND | ND | ND | 535.96 | 615.18 | 578.68 |
|  | Carukiidae | *Carukia barnesi* | 20 | Palm Cove, QLD, Australia | ND | ND | ND | 130.03 | 322.68 | 223.72 |
|  |  | *Malo maxima* | 20 | Port Douglas, QLD, Australia | 9.50 | 15.00 | 11.64 | 304.62 | 433.59 | 358.38 |
|  |  | *Morbakka fenneri* | 11 | Townsville, QLD, Australia | 40.00 | 90.00 | 58.64 | 501.39 | 647.19 | 551.34 |
|  | Carybdeidae | *Carybdea rastonii* | 9 | Marino Rocks, SA, Australia | 3.60 | 8.80 | 5.87 | 302.48 | 386.72 | 350.23 |
|  |  | *Carybdea xaymacana* | 17 | Busselton, WA [*n* (10)]; Townsville, QLD [*n* (7)], Australia | 2.80 | 16.00 | 6.98 | 250.38 | 436.22 | 326.45 |
|  | Tripedaliidae | *Copula sivickisi* | 20 | Townsville, QLD, Australia | 3.00 | 6.00 | 4.47 | 279.49 | 375.42 | 326.95 |
|  |  | *Tripedalia binata* | 12 | Darwin, NT [*n* (9)]; Cape York, QLD [*n* (3)], Australia | 3.80 | 13.00 | 7.21 | 364.57 | 465.08 | 397.58 |
|  |  | *Tripedalia cystophora* | 20 | Lab Culture, Denmark | 3.00 | 5.50 | 4.09 | 223.18 | 315.06 | 267.79 |
| Chirodropida | Chirodropidae | *Chironex fleckeri* | 20 | Cape York, QLD, Australia | 20.00 | 130.00 | 94.75 | 489.94 | 920.24 | 763.14 |
|  | Chiropsalmidae | *Chiropsella bart* | 4 | Gove, NT, Australia | ND | ND | ND | 530.01 | 597.54 | 568.40 |
|  |  | *Chiropsella bronzie* | 20 | Port Douglas, QLD, Australia | 20.00 | 40.00 | 27.02 | 382.91 | 495.66 | 439.90 |

**S1 Table B.** Number of harmonics required to gain > 99% shape power from Elliptical Fourier Analysis for statolith proximal, oral and lateral faces of 12 cubozoan species. Min.; Minimum, Max.; Maximum

|  |  | Proximal | | | Oral | | | Lateral | | |
| --- | --- | --- | --- | --- | --- | --- | --- | --- | --- | --- |
| Species | *n* | Min. | Max. | Mean | Min. | Max. | Mean | Min. | Max. | Mean |
| *Alatina moseri* | 12 | 5 | 12 | 7.75 | 5 | 12 | 7.75 | 5 | 12 | 7.75 |
| *Carukia barnesi* | 20 | 5 | 14 | 8.80 | 5 | 14 | 8.80 | 5 | 14 | 8.80 |
| *Malo maxima* | 20 | 6 | 14 | 9.35 | 6 | 18 | 12.65 | 8 | 15 | 10.55 |
| *Morbakka fenneri* | 11 | 7 | 13 | 9.55 | 7 | 18 | 13.36 | 6 | 11 | 8.73 |
| *Carybdea rastonii* | 9 | 4 | 9 | 4.78 | 6 | 14 | 8.89 | 6 | 13 | 9.22 |
| *Carybdea xaymacana* | 17 | 4 | 7 | 5.00 | 7 | 15 | 11.00 | 6 | 14 | 9.29 |
| *Copula sivickisi* | 20 | 4 | 6 | 4.85 | 4 | 7 | 5.45 | 7 | 13 | 10.40 |
| *Tripedalia binata* | 12 | 4 | 8 | 5.33 | 5 | 14 | 9.00 | 9 | 15 | 11.83 |
| *Tripedalia cystophora* | 20 | 5 | 10 | 6.75 | 10 | 16 | 13.20 | 8 | 18 | 12.60 |
| *Chironex fleckeri* | 20 | 4 | 8 | 6.60 | 5 | 11 | 6.60 | 7 | 14 | 9.85 |
| *Chiropsella bart* | 4 | 5 | 11 | 7.75 | 10 | 12 | 11.25 | 10 | 16 | 13.00 |
| *Chiropsella bronzie* | 20 | 5 | 7 | 5.65 | 6 | 16 | 10.95 | 7 | 14 | 10.20 |

**S1 Table C.** Tukey HSD Multiple Comparisons for ANOVA of statolith Length: Width among species for (*a*) proximal, (*b*) oral and (*c*) lateral faces. NS = No significant difference, * = *p* < 0.05, ** = *p* < 0.01, *** = *p* < 0.001 *(Corresponds to Fig 2 main text)*

| (*a*) Proximal face | | *Alatina* *moseri* | *Carukia barnesi* | *Malo maxima* | *Morbakka* *fenneri* | *Carybdea rastonii* | *Carybdea xaymacana* | *Copula sivickisi* | *Tripedalia binata* | *Tripedalia cystophora* | *Chironex fleckeri* | *Chiropsella bart* | *Chiropsella bronzie* |
| --- | --- | --- | --- | --- | --- | --- | --- | --- | --- | --- | --- | --- | --- |
| Family | Species |  |  |  |  |  |  |  |  |  |  |  |  |
| Alatinidae | *Alatina moseri* | NS |  |  |  |  |  |  |  |  |  |  |  |
| Carukiidae | *Carukia barnesi* | NS | NS |  |  |  |  |  |  |  |  |  |  |
|  | *Malo maxima* | * | NS | NS |  |  |  |  |  |  |  |  |  |
|  | *Morbakka fenneri* | * | NS | NS | NS |  |  |  |  |  |  |  |  |
| Carybdeidae | *Carybdea rastonii* | *** | *** | *** | *** | NS |  |  |  |  |  |  |  |
|  | *Carybdea xaymacana* | *** | *** | *** | *** | NS | NS |  |  |  |  |  |  |
| Tripedaliidae | *Copula sivickisi* | *** | *** | *** | *** | *** | *** | NS |  |  |  |  |  |
|  | *Tripedalia binata* | *** | *** | *** | *** | * | ** | *** | NS |  |  |  |  |
|  | *Tripedalia cystophora* | NS | *** | *** | *** | ** | *** | *** | *** | NS |  |  |  |
| Chirodropidae | *Chironex fleckeri* | *** | *** | *** | *** | NS | * | *** | NS | *** | NS |  |  |
| Chiropsalmidae | *Chiropsella bart* | NS | ** | ** | ** | NS | NS | *** | *** | NS | *** | NS |  |
|  | *Chiropsella bronzie* | NS | ** | *** | ** | *** | *** | *** | *** | NS | *** | NS | NS |

**S1 Table C** continued

| (*b*) Oral face | | *Alatina* *moseri* | *Carukia barnesi* | *Malo maxima* | *Morbakka* *fenneri* | *Carybdea rastonii* | *Carybdea xaymacana* | *Copula sivickisi* | *Tripedalia binata* | *Tripedalia cystophora* | *Chironex fleckeri* | *Chiropsella bart* | *Chiropsella bronzie* |
| --- | --- | --- | --- | --- | --- | --- | --- | --- | --- | --- | --- | --- | --- |
| Family | Species |  |  |  |  |  |  |  |  |  |  |  |  |
| Alatinidae | *Alatina moseri* | NS |  |  |  |  |  |  |  |  |  |  |  |
| Carukiidae | *Carukia barnesi* | NS | NS |  |  |  |  |  |  |  |  |  |  |
|  | *Malo maxima* | NS | NS | NS |  |  |  |  |  |  |  |  |  |
|  | *Morbakka fenneri* | NS | NS | NS | NS |  |  |  |  |  |  |  |  |
| Carybdeidae | *Carybdea rastonii* | ** | ** | *** | *** | NS |  |  |  |  |  |  |  |
|  | *Carybdea xaymacana* | NS | * | ** | ** | NS | NS |  |  |  |  |  |  |
| Tripedaliidae | *Copula sivickisi* | *** | *** | *** | *** | *** | *** | NS |  |  |  |  |  |
|  | *Tripedalia binata* | *** | *** | *** | *** | *** | *** | *** | NS |  |  |  |  |
|  | *Tripedalia cystophora* | *** | *** | *** | *** | NS | NS | *** | *** | NS |  |  |  |
| Chirodropidae | *Chironex fleckeri* | *** | *** | *** | *** | *** | *** | *** | ** | *** | NS |  |  |
| Chiropsalmidae | *Chiropsella bart* | ** | ** | *** | ** | NS | NS | *** | ** | NS | *** | NS |  |
|  | *Chiropsella bronzie* | * | ** | *** | ** | NS | NS | *** | *** | NS | *** | NS | NS |

**S1 Table C** continued

| (*c*) Lateral face | | *Alatina* *moseri* | *Carukia barnesi* | *Malo maxima* | *Morbakka* *fenneri* | *Carybdea rastonii* | *Carybdea xaymacana* | *Copula sivickisi* | *Tripedalia binata* | *Tripedalia cystophora* | *Chironex fleckeri* | *Chiropsella bart* | *Chiropsella bronzie* |
| --- | --- | --- | --- | --- | --- | --- | --- | --- | --- | --- | --- | --- | --- |
| Family | Species |  |  |  |  |  |  |  |  |  |  |  |  |
| Alatinidae | *Alatina moseri* | NS |  |  |  |  |  |  |  |  |  |  |  |
| Carukiidae | *Carukia barnesi* | *** | NS |  |  |  |  |  |  |  |  |  |  |
|  | *Malo maxima* | *** | NS | NS |  |  |  |  |  |  |  |  |  |
|  | *Morbakka fenneri* | *** | NS | * | NS |  |  |  |  |  |  |  |  |
| Carybdeidae | *Carybdea rastonii* | *** | NS | NS | NS | NS |  |  |  |  |  |  |  |
|  | *Carybdea xaymacana* | *** | NS | NS | NS | NS | NS |  |  |  |  |  |  |
| Tripedaliidae | *Copula sivickisi* | *** | *** | *** | NS | NS | *** | NS |  |  |  |  |  |
|  | *Tripedalia binata* | *** | *** | *** | NS | * | *** | NS | NS |  |  |  |  |
|  | *Tripedalia cystophora* | *** | ** | *** | NS | NS | ** | NS | NS | NS |  |  |  |
| Chirodropidae | *Chironex fleckeri* | *** | * | ** | NS | NS | * | NS | NS | NS | NS |  |  |
| Chiropsalmidae | *Chiropsella bart* | *** | *** | *** | * | *** | *** | NS | NS | NS | * | NS |  |
|  | *Chiropsella bronzie* | *** | *** | *** | ** | *** | *** | NS | NS | * | ** | NS | NS |

**S1 Table D.** Canonical Discriminant Analysis classifications for statolith Length: Width ratios for proximal + oral + lateral faces (bold = percent of samples correctly classified). Percentages (%) are rounded to whole numbers *(Corresponds to Fig 3 main text)*

|  |  |  | % correct classification | Species classified to following Jackknifed classification  (% of samples) | | | | | | | | | | | |
| --- | --- | --- | --- | --- | --- | --- | --- | --- | --- | --- | --- | --- | --- | --- | --- |
| Family | Species | *n* |  | *Alatina moseri* | *Carukia barnesi* | *Malo maxima* | *Morbakka fenneri* | *Carybdea rastonii* | *Carybdea xaymacana* | *Copula sivickisi* | *Tripedalia binata* | *Tripedalia cystophora* | *Chironex fleckeri* | *Chiropsella bart* | *Chiropsella bronzie* |
| Alatinidae | *Alatina moseri* | 12 | **75** | **58** | 25 | 17 |  |  |  |  |  |  |  |  |  |
| Carukiidae | *Carukia barnesi* | 20 | **45** | 15 | **45** | 30 | 5 |  |  |  |  | 5 |  |  |  |
|  | *Malo maxima* | 20 | **55** | 5 | 35 | **55** | 5 |  |  |  |  |  |  |  |  |
|  | *Morbakka fenneri* | 11 | **18** |  | 18 | 36 | **18** |  |  |  |  |  |  |  | 27 |
| Carybdeidae | *Carybdea rastonii* | 9 | **11** |  |  |  |  |  | 78 |  |  | 22 |  |  |  |
|  | *Carybdea xaymacana* | 17 | **71** | 6 |  |  |  | 6 | **71** |  |  | 12 | 6 |  |  |
| Tripedaliidae | *Copula sivickisi* | 20 | **85** |  |  |  |  |  |  | **80** | 10 |  | 10 |  |  |
|  | *Tripedalia binata* | 12 | **92** |  |  |  |  |  |  |  | **83** |  | 17 |  |  |
|  | *Tripedalia cystophora* | 20 | **60** |  | 5 |  |  |  | 10 |  | 5 | **55** |  |  | 25 |
| Chirodropidae | *Chironex fleckeri* | 20 | **75** |  |  |  |  |  |  |  | 15 | 15 | **70** |  |  |
| Chiropsalmidae | *Chiropsella bart* | 4 | **0** |  |  |  |  |  |  |  |  | 25 |  |  | 75 |
|  | *Chiropsella bronzie* | 20 | **70** |  | 10 |  |  |  |  |  |  | 25 |  |  | **65** |

**S1 Table E.** Least squares regression results for dependence of statolith shape on statolith size. * = *p* < 0.05

| Species | *n* |  | R^2^ |
| --- | --- | --- | --- |
| *Alatina* *moseri* | 12 |  | 0.037 |
| *Carukia barnesi* | 20 |  | 0.050 |
| *Malo maxima* | 20 |  | 0.278 * |
| *Morbakka fenneri* | 11 |  | 0.270 |
| *Carybdea rastonii* | 9 |  | 0.112 |
| *Carybdea xaymacana* | 17 |  | 0.001 |
| *Copula sivickisi* | 20 |  | 0.038 |
| *Tripedalia binata* | 12 |  | 0.035 |
| *Tripedalia cystophora* | 20 |  | 0.000 |
| *Chironex fleckeri* | 20 |  | 0.000 |
| *Chiropsella bart* | 4 |  | 0.631 |
| *Chiropsella bronzie* | 20 |  | 0.027 |

**S1 Appendix A.** Measuring Length: Width ratios in R

### Measuring statolith Length and Width using locator landmarks ###

#load packages

library (pixmap)

library (rtiff)

#import image, by choosing image file from folder

statolith <- readTiff(choose.files(), reduce=0)

#convert to greyimage

statolith <- as(statolith, "pixmapGrey")

str(statolith)

plot(statolith)

#calibrating to scale

#using locator function

a <- locator (2, type="p", pch=3)

#use mouse to click ends of 100um scale bar

scale100um <- sqrt(sum(diff(a$x)^2+diff(a$y)^2))

#mark landmarks i - iv using locator with mouse, then calibrate to scale

d <- locator (4, type="p", pch=21, bg="white")

d <- rbind (d$x, d$y)/scale100um

#name landmark coordinates

i <- c(d[1,1], d[2,1])

ii <- c(d[1,2], d[2,2])

iii <- c(d[1,3], d[2,3])

iv <- c(d[1,4], d[2,4])

#use ild function to measure distances between landmarks

ild <- function(i,ii) {sqrt(sum((i-ii)^2))}

#measure length & width giving output in microns

length <- (ild(i,ii))*100; length

width <- (ild(iii,iv))*100; width

Calibration was achieved by clicking either end of the scale bar which then made it possible to obtain inter-landmark distances using the Cartesian coordinates of the scale. Then locator was used to mark landmarks i, ii, iii and iv on the silhouette (see S1 Fig A). For the statolith proximal face length (i-ii) was the statolith’s longest axis and width (iii-iv) was the midpoint of cleavage (iii) to the opposite edge (iv) perpendicular to i-ii. Oral face length (i-ii) was the same as for proximal face length and width (iii-iv) was from the point of extremity (iii) to the opposite edge (iv) perpendicular to i-ii. Lateral face length (i-ii) was the longest axis from the point of extremity if present and width (iii-iv) was perpendicular at the widest point or from cleft (iii) if present to opposite edge (iv) perpendicular to i-ii. Length and width distances were then calculated using the ild function developed by Claude (2008) which computes the distance between any two landmarks as the square root of the sum of the squared differences between each coordinate.


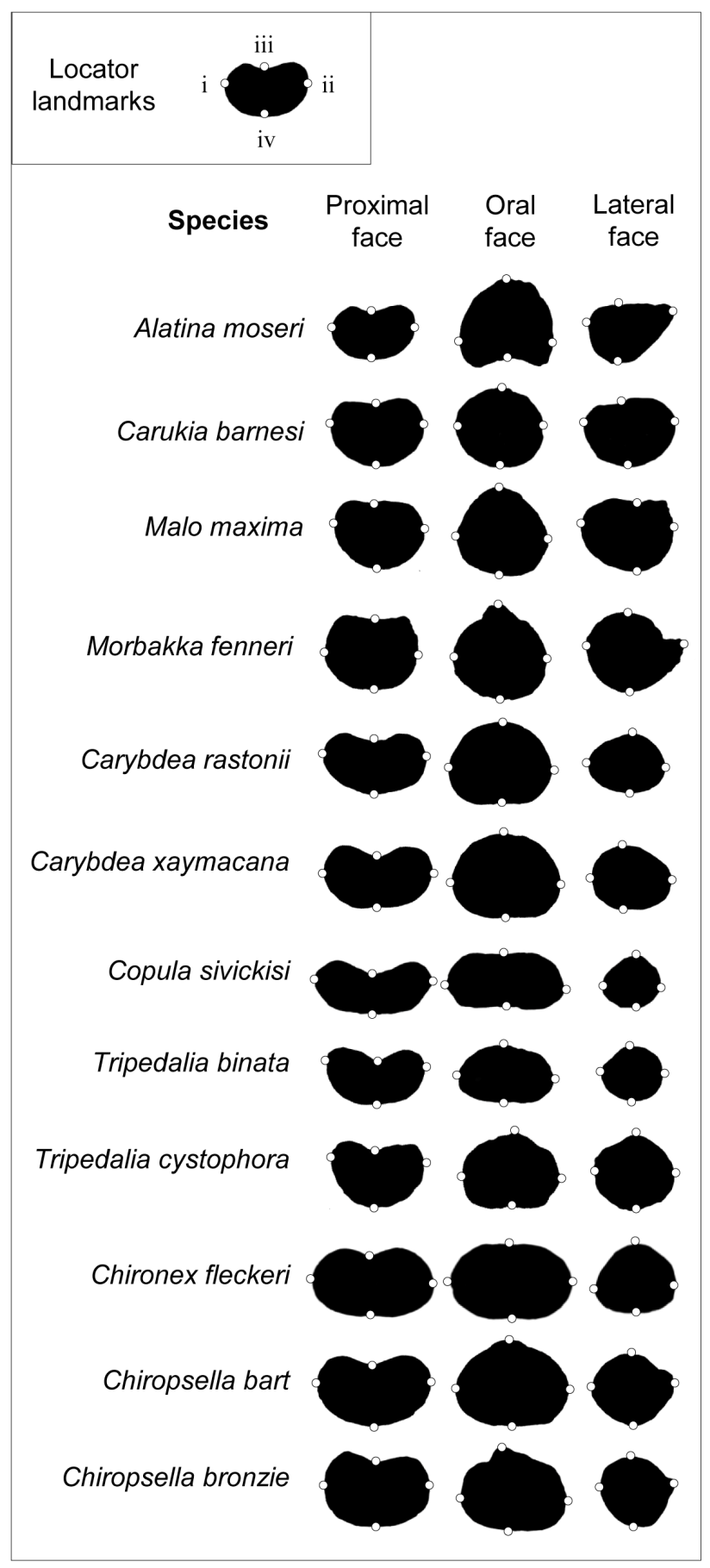


**S1 Fig A.** Locator landmark positions for proximal, oral and lateral statolith faces of 12 cubozoan species used for Length: Width measurements

**S1 Appendix B.** Performing Elliptical Fourier Analysis in R

### Using Momocs for Normalised Elliptical Fourier Analysis of statolith silhouettes ###

##load Momocs package

library(Momocs)

#import .jpg image by choosing file from folder and convert to coordinates of outline

statolith <- import.jpg(choose.files())

#create Coo object using outline coordinates

coostat <- Coo(statolith)

#estimate number of Fourier harmonics to retain by >99% total cumulative power

hpow(coostat)

#perform elliptical fourier analysis (normalised) on Coo object, for 20 harmonics, 300 smoothing iterations

ef <- eFourier(coostat, 20, 300, T, F)

ef

#write table of harmonic coefficients to clipboard for export to excel

write.table(ef@coe, "clipboard", sep="\t", col.names=NA)

#plot outline to check correct silhouette

coo.plot(coostat)
